# Supplementary material for: Indoor and outdoor fine particulate matter and carbon monoxide concentrations in homes of infants in Nairobi, Kenya
Source: PLOS Glob Public Health. 2026 Apr 6;6(4):e0006202. doi: 10.1371/journal.pgph.0006202 (PMC13052846; doi:10.1371/journal.pgph.0006202)
Supplement: S1 Table — (DOCX) [file pgph.0006202.s001.docx]

**Indoor and outdoor fine particulate matter and carbon monoxide concentrations in homes of infants in Nairobi, Kenya**

**Supporting information**

**S1 Table. Select demographic and household characteristics at enrollment of air sampling participants and the full ABC cohort.**

|  |  | **Air monitoring subsample**  **(n = 48)** | | **ABC cohort**  **(N = 400)** | |
| --- | --- | --- | --- | --- | --- |
| **Characteristic** | **Detail** | **Frequency or median** | **Percent or IQR** | **Frequency or median** | **Percent or IQR** |
| **Socio-demographics at enrollment** | | | | | |
| Maternal age, years | Median | 27 | 24-31 | 26 | 23-31 |
| Maternal education level | Primary | 8 | 16.7 | 104^a^ | 26.0 |
|  | Secondary | 32 | 66.7 | 232 | 58.0 |
|  | University or college | 8 | 16.7 | 62 | 15.5 |
| Maternal employment status | Homemaker or none | 30 | 62.5 | 216 | 54.0 |
|  | Daily wage | 6 | 12.5 | 45 | 11.3 |
|  | Small business | 6 | 12.5 | 68 | 17.0 |
|  | Monthly salary | 4 | 8.3 | 36 | 9.0 |
| Monthly household rent (KES) | Median (renters only) | 4,000  (n = 41) | 3,000-5,500 | 3,500  (n = 330) | 2,500-5,000 |
| **Household characteristics** | | | | | |
| Type of housing^b^ | Small bungalow (single unit) | 1 | 2.1 | N/A | N/A |
|  | Multi-unit dwelling (flats) | 47 | 97.9 | N/A | N/A |
| Number of persons in household | 2-4 | 34 | 70.8 | 337 | 84.3 |
|  | 5-8 | 14 | 29.2 | 63^c^ | 15.8 |
| Number of rooms | 1 | 24 | 50.0 | 231 | 57.8 |
|  | 2 | 16 | 33.3 | 120 | 30.0 |
|  | 3 or more | 8 | 16.7 | 49 | 12.3 |
| Persons per room |  | 3 | 2-4 | 2 | 1.5-3.0 |
| Water source | Borehole/rainwater/ river | 0 | 0 | 2 | 0.5 |
|  | Water vendor | 2 | 4.2 | 10 | 2.5 |
|  | Piped water outside house | 40 | 83.3 | 349 | 87.3 |
|  | Piped water inside house | 8 | 16.7 | 49 | 12.3 |
| Type of toilet | Pit latrine | 0 | 0 | 12 | 3.0 |
|  | Flush | 48 | 100 | 388 | 97.0 |
| Shared toilet^d^ | Yes | 42 | 87.5 | N/A | N/A |
|  | No | 6 | 12.5 | N/A | N/A |
| Roof material | Metal sheets | 33 | 68.8 | 338 | 84.5 |
|  | Concrete | 15 | 31.3 | 58 | 14.5 |
| Wall material | Metal sheets | 1 | 2.1 | 23 | 5.8 |
|  | Stone | 47 | 97.9 | 375 | 93.8 |
| Floor material | Cemented | 34 | 70.8 | 299 | 74.8 |
|  | Ceramic tiles | 15 | 31.3 | 84 | 21.0 |
| **House ventilation features** | | | | | |
| Number of external windows | 0 | 1 | 2.1 | 10 | 2.5 |
|  | 1 | 20 | 41.7 | 226 | 56.5 |
|  | 2 | 19 | 39.6 | 111 | 27.8 |
|  | 3 or more | 8 | 16.7 | 23 | 5.8 |
| Number of external doors | 1 | 41^e^ | 85.4 | 359 | 89.8 |
|  | 2 or more | 6 | 12.5 | 41 | 10.3 |
| **Household behaviors related to air pollution** | | | | | |
| Rug or carpet floor covering | No | 22 | 45.8 | 263 | 65.8 |
|  | Yes | 26 | 54.2 | 137 | 34.3 |
| Fuels used by the household indoors | Wood | 1 | 2.1 | 8 | 2.0 |
|  | Charcoal | 1 | 2.1 | 44 | 11.0 |
|  | Kerosene | 18 | 37.5 | 216 | 54.0 |
|  | Ethanol (Koko fuel) | 15 | 31.3 | 70 | 17.5 |
|  | Liquefied petroleum gas | 40 | 83.3 | 286 | 71.5 |
|  | Electricity | 5 | 10.4 | 19 | 4.8 |

IQR = interquartile range. N/A = not available. ^a^Includes 1 pre-primary and 2 never went to school. ^b^Technician observed at air sampling visit; not collected at enrollment. ^c^Includes one home with 9 people. ^d^Collected at air sampling visit; not collected at enrollment. ^e^1 missing response.
