# Supplementary material for: Indoor and outdoor fine particulate matter and carbon monoxide concentrations in homes of infants in Nairobi, Kenya
Source: PLOS Glob Public Health. 2026 Apr 6;6(4):e0006202. doi: 10.1371/journal.pgph.0006202 (PMC13052846; doi:10.1371/journal.pgph.0006202)
Supplement: S2 Table — (DOCX) [file pgph.0006202.s002.docx]

**Indoor and outdoor fine particulate matter and carbon monoxide concentrations in homes of infants in Nairobi, Kenya**

**Supporting information**

**S2 Table. Tests of differences in indoor PM_2.5_ concentrations by selected household characteristics and combustion activities during air sampling in a subsample of 39 homes.**

| **Characteristic/ activity** | **Detail** | **Count or median (IQR)** | **Geo. mean (ug/m^3^) (GSD) PM_2.5_** | **Test statistic** | ***p*-value** |
| --- | --- | --- | --- | --- | --- |
| Number of persons in household | 2-4 | 25 | 41.7 (1.8) | t = -0.77 | 0.45 |
|  | 5-8 | 14 | 97.8 (145.2) |  |  |
| Number of rooms | 1 | 20 | 39.0. (1.7) | t = -1.23 | 0.23 |
|  | 2-4 | 19 | 52.6 (2.5) |  |  |
| Kitchen volume | m^3^ | 22.4 (12.0) |  | F = 3.31 | 0.08 |
| Total external windows and doors | 1-2 | 18 | 38.6 (1.8) | t = -1.23 | 0.23 |
|  | 3-7 | 21 | 51.5 (2.4) |  |  |
| Rug or carpet floor covering | No | 17 | 49.2 (1.8) | t = 0.66 | 0.51 |
|  | Yes | 22 | 42.2 (2.4) |  |  |
| Kerosene use | No | 35 | 43.8 (2.2) | perm. test = 5.72 | 0.68 |
|  | Yes | 4 | 58.1 (1.4) |  |  |
| LPG use | No | 15 | 35.9 (1.8) | t = -1.63 | 0.11 |
|  | Yes | 24 | 52.0 (2.3) |  |  |
| Ethanol fuel use | No | 30 | 48.9 (2.2) | t = 1.43 | 0.17 |
|  | Yes | 9 | 34.4 (1.8) |  |  |
| Electricity only - no other fuels | No | 36 | 47.9 (2.1) | perm. test = 47.24 | 0.01 |
|  | Yes | 3 | 21.8 (1.3) |  |  |
| Cigarette and/or marijuana smoke in the home | No | 35 | 46.1 (2.2) | perm. test = 28.23 | 0.31 |
|  | Yes | 4 | 37.4 (1.6) |  |  |
| Burning mosquito repellent | No | 35 | 41.8 (1.9) | perm. test = -114.09 | 0.96 |
|  | Yes | 4 | 88.8 (3.4) |  |  |
| Burning candles | No | 31 | 43.0 (2.1) | t = -0.75 | 0.47 |
|  | Yes | 8 | 54.2 (2.2) |  |  |

IQR, interquartile range. Geo. mean, geometric mean. GSD, geometric standard deviation. Perm. test, test statistic of two-sample permutation test (for cell counts < 5).
