# Supplementary material for: Indoor and outdoor fine particulate matter and carbon monoxide concentrations in homes of infants in Nairobi, Kenya
Source: PLOS Glob Public Health. 2026 Apr 6;6(4):e0006202. doi: 10.1371/journal.pgph.0006202 (PMC13052846; doi:10.1371/journal.pgph.0006202)
Supplement: S3 Table — (DOCX) [file pgph.0006202.s003.docx]

**Indoor and outdoor fine particulate matter and carbon monoxide concentrations in homes of infants in Nairobi, Kenya**

**Supporting information**

**S3 Table. Tests of differences in outdoor PM_2.5_ concentrations by participant reported exposure to air pollution from selected outdoor sources during air sampling in a subsample of 39 homes.**

| **Characteristic/ activity** | **Detail** | **Count or median (IQR)** | **Geo. mean (ug/m^3^) (GSD) PM_2.5_** | **Test statistic** | ***p*-value** |
| --- | --- | --- | --- | --- | --- |
| Smoke from dumpsite (within 1 km) | No | 24 | 21.1 (2.0) | t = -0.64 | 0.53 |
|  | Yes | 15 | 25.6 (1.6) |  |  |
| Smoke from rubbish burning (within 1 km) | No | 10 | 21.2 (1.3) | t = -1.87 | 0.07 |
|  | Yes | 29 | 23.3 (2.0) |  |  |
| Dust from unpaved roads (within 1 km) | No | 5 | 26.7 (1.9) | t = 0.61 | 0.57 |
|  | Yes | 34 | 22.2 (1.8) |  |  |
| Construction dust (within 1 km) | No | 27 | 23.2 (2.0) | t = 1.02 | 0.31 |
|  | Yes | 12 | 21.6 (1.5) |  |  |

IQR, interquartile range. Geo. mean, geometric mean. GSD, geometric standard deviation.
