# Supplementary material for: Indoor and outdoor fine particulate matter and carbon monoxide concentrations in homes of infants in Nairobi, Kenya
Source: PLOS Glob Public Health. 2026 Apr 6;6(4):e0006202. doi: 10.1371/journal.pgph.0006202 (PMC13052846; doi:10.1371/journal.pgph.0006202)
Supplement: S4 Table — (DOCX) [file pgph.0006202.s004.docx]

**Indoor and outdoor fine particulate matter and carbon monoxide concentrations in homes of infants in Nairobi, Kenya**

**Supporting information**

**S4 Table. Tests of differences in indoor PM_2.5_ concentrations by selected household characteristics and combustion activities during air sampling in a subsample of 36 homes with complete indoor and outdoor PM_2.5_ data, by PM_2.5_ indoor/outdoor (I/O) ratio group.**

| **Characteristic/ activity** | **Detail** | **Count or median (IQR)** | **Geo. mean (ug/m^3^) (GSD) PM_2.5_** | **Test statistic** | ***p*-value** |
| --- | --- | --- | --- | --- | --- |
| **Homes with PM_2.5_ I/O ratio ≤ median (1.6)** | | | | | |
| Number of persons in household | 2-4 | 11 | 32.6 (1.7) | t = -0.01 | 0.99 |
|  | 5-8 | 7 | 32.7 (1.6) |  |  |
| Number of rooms | 1 | 8 | 31.9 (1.8) | t = -0.17 | 0.87 |
|  | 2-4 | 10 | 33.3 (1.6) |  |  |
| Kitchen volume | m^3^ | 26.0 (15.7) |  | F = 1.12 | 0.31 |
| Total external windows and doors | 1-2 | 6 | 29.1 (2.0) | t = -0.57 | 0.58 |
|  | 3-7 | 12 | 34.7 (1.5) |  |  |
| Rug or carpet floor covering | No | 7 | 40.0 (1.7) | t = 1.35 | 0.20 |
|  | Yes | 11 | 28.7 (1.6) |  |  |
| Kerosene use | No | 17 | 32.0 (1.7) | -- | -- |
|  | Yes | 1 | 46.2 |  |  |
| LPG use | No | 9 | 29.5 (1.8) | t = -0.87 | 0.40 |
|  | Yes | 9 | 36.2 (1.4) |  |  |
| Ethanol fuel use | No | 12 | 33.5 (1.5) | t = 0.24 | 0.82 |
|  | Yes | 6 | 31.2 (2.0) |  |  |
| Electricity only - no other fuels | No | 16 | 34.8 (1.6) | -- | -- |
|  | Yes | 2 | 20.0 (1.4) |  |  |
| Cigarettes and/or marijuana smoke in the home | No | 15 | 31.5 (1.6) | perm. test = -54.87 | 0.87 |
|  | Yes | 3 | 39.4 (1.8) |  |  |
| Burning mosquito repellent | No | 16 | 31.7 (1.7) | -- | -- |
|  | Yes | 2 | 42.2 (1.4) |  |  |
| Burning candles | No | 17 | 31.7 (1.6) | -- | -- |
|  | Yes | 1 | 54.1 |  |  |
| **Homes with PM_2.5_ I/O ratio > median (1.6)** | | | | | |
| Number of persons in household | 2-4 | 11 | 52.9 (1.8) | t = -0.93 | 0.38 |
|  | 5-8 | 7 | 82.4 (3.2) |  |  |
| Number of rooms | 1 | 9 | 45.3 (1.6) | t = -1.71 | 0.12 |
|  | 2-4 | 9 | 87.3 (2.9) |  |  |
| Kitchen volume | m^3^ | 21.7 (7.7) |  | F = 2.39 | 0.1 |
| Total external windows and doors | 1-2 | 9 | 45.3 (1.6) | t = -1.71 | 0.11 |
|  | 3-7 | 9 | 87.3 (2.9) |  |  |
| Rug or carpet floor covering | No | 9 | 54.0 (1.9) | t = -0.74 | 0.47 |
|  | Yes | 9 | 73.2 (2.9) |  |  |
| Kerosene use | No | 15 | 62.9 (2.6) | perm. test = -88.67 | 0.96 |
|  | Yes | 3 | 62.7 (1.5) |  |  |
| LPG use | No | 5 | 53.2 (1.6) | t = -0.66 | 0.52 |
|  | Yes | 13 | 67.1 (2.7) |  |  |
| Ethanol fuel use | No | 16 | 64.7 (2.5) | -- | -- |
|  | Yes | 2 | 50.1 (1.5) |  |  |
| Electricity only - no other fuels | No | 17 | 66.3 (2.4) | -- | -- |
|  | Yes | 1 | 25.8 |  |  |
| Cigarette and/or marijuana smoke in the home | No | 11 | 65.4 (2.4) | -- | -- |
|  | Yes | 1 | 32.2 |  |  |
| Burning mosquito repellent | No | 16 | 54.9 (2.1) | -- | -- |
|  | Yes | 2 | 186.7 (4.3) |  |  |
| Burning candles | No | 12 | 64.4 (2.4) | 0.16 | 0.88 |
|  | Yes | 6 | 60.0 (2.4) |  |  |

IQR, interquartile range. Geo. mean, geometric mean. GSD, geometric standard deviation. Perm. test, test statistic of two-sample permutation test (for cell counts < 5). Note: test statistics not calculated for groups with < 3 observations.
