# Supplementary material for: Indoor and outdoor fine particulate matter and carbon monoxide concentrations in homes of infants in Nairobi, Kenya
Source: PLOS Glob Public Health. 2026 Apr 6;6(4):e0006202. doi: 10.1371/journal.pgph.0006202 (PMC13052846; doi:10.1371/journal.pgph.0006202)
Supplement: S5 Table — (DOCX) [file pgph.0006202.s005.docx]

**Indoor and outdoor fine particulate matter and carbon monoxide concentrations in homes of infants in Nairobi, Kenya**

**Supporting information**

**S5 Table. Tests of differences in indoor PM_2.5_ concentrations by participant reported exposure to air pollution from selected outdoor sources during air sampling in a subsample of 36 homes with complete indoor and outdoor PM_2.5_ data, by PM_2.5_ I/O ratio group.**

| **Characteristic/ activity** | **Detail** | **Count or median (IQR)** | **Geo. mean (ug/m^3^) (GSD) PM_2.5_** | **Test statistic** | ***p*-value** |
| --- | --- | --- | --- | --- | --- |
| **Homes with PM_2.5_ I/O ratio ≤ median (1.6)** | | | | | |
| Outdoor smoke from our own or neighbors’ cooking | No | 15 | 35.0 (1.5) | perm. test = 9.97 | 0.17 |
|  | Yes | 3 | 23.1 (2.1) |  |  |
| Smoke from dumpsite (within 1 km) | No | 12 | 31.1 (1.7) | t = -1.53 | 0.17 |
|  | Yes | 6 | 36.1 (1.4) |  |  |
| Smoke from rubbish burning (within 1 km) | No | 3 | 19.3 (1.3) | perm. test = -19.93 | 0.98 |
|  | Yes | 15 | 36.3 (1.6) |  |  |
| Dust from unpaved roads (within 1 km) | No | 2 | 51.3 (1.4) | -- | -- |
|  | Yes | 16 | 30.9 (1.6) |  |  |
| Construction dust (within 1 km) | No | 12 | 35.3(1.6) | t = 0.72 | 0.48 |
|  | Yes | 6 | 33.5 (18.9) |  |  |
| **Homes with PM_2.5_ I/O ratio > median (1.6)** | | | | | |
| Outdoor smoke from our own or neighbors’ cooking | No | 14 | 69.7 (2.5) | perm. test = 62.55 | 0.22 |
|  | Yes | 4 | 43.9 (1.7) |  |  |
| Smoke from dumpsite (within 1 km) | No | 11 | 45.3 (1.9) | t = -1.53 | 0.17 |
|  | Yes | 7 | 105.4 (2.6) |  |  |
| Smoke from rubbish burning (within 1 km) | No | 5 | 85.5 (2.9) | t = 0.74 | 0.49 |
|  | Yes | 13 | 55.9 (2.2) |  |  |
| Dust from unpaved roads (within 1 km) | No | 3 | 64.8(2.8) | perm. test = -4.14 | 0.42 |
|  | Yes | 15 | 62.5 (2.4) |  |  |
| Construction dust (within 1 km) | No | 13 | 79.6 (2.5) | t = 3.21 | 0.006 |
|  | Yes | 5 | 35.1 (6.3) |  |  |

IQR, interquartile range. Geo. mean, geometric mean. GSD, geometric standard deviation. Perm. test, test statistic of two-sample permutation test (for cell counts < 5). Note: test statistics not calculated for groups with < 3 observations.
