# Supplementary material for: Indoor and outdoor fine particulate matter and carbon monoxide concentrations in homes of infants in Nairobi, Kenya
Source: PLOS Glob Public Health. 2026 Apr 6;6(4):e0006202. doi: 10.1371/journal.pgph.0006202 (PMC13052846; doi:10.1371/journal.pgph.0006202)
Supplement: S6 Table — (DOCX) [file pgph.0006202.s006.docx]

**Indoor and outdoor fine particulate matter and carbon monoxide concentrations in homes of infants in Nairobi, Kenya**

**Supporting information**

**S6 Table. Tests of differences in 24 h mean indoor CO concentrations by selected household characteristics and combustion activities during air sampling in a subsample of 47 homes.**

| **Characteristic/ activity** | **Detail** | **Count or median (IQR)** | **Geo. mean (ppm) (GSD) CO** | **Test statistic** | ***p*-value** |
| --- | --- | --- | --- | --- | --- |
| Number of persons in household | 2-4 | 33 | 0.7 (2.0) | t = 0.71 | 0.49 |
|  | 5-8 | 14 | 1.2 (1.9) |  |  |
| Number of rooms | 1 | 23 | 1.8 (2.3) | t = 0.79 | 0.43 |
|  | 2-4 | 24 | 0.6 (2.0) |  |  |
| Kitchen volume | m^3^ | 24.6 (12.1) |  | F = 0.34 | 0.56 |
| Total external windows and doors | 1-2 | 20 | 2.0 (3.7) | t = -0.27 | 0.79 |
|  | 3-7 | 27 | 0.7 (1.9) |  |  |
| Kerosene use | No | 42 | 0.6 (1.9) | t = -3.90 | 0.0003 |
|  | Yes | 5 | 3.3 (3.1) |  |  |
| LPG use | No | 16 | 2.2 (4.4) | t = 0.18 | 0.86 |
|  | Yes | 31 | 0.5 (1.4) |  |  |
| Ethanol fuel use | No | 38 | 0.6 (1.9) | t = 1.02 | 0.33 |
|  | Yes | 9 | 3.7 (6.0) |  |  |
| Electricity only - no other fuels | No | 44 | 0.8 (2.2) | perm. test = 2.44 | 0.14 |
|  | Yes | 3 | 0.0 (0.9) |  |  |
| Cigarette and/or marijuana smoke in the home | No | 41 | 0.9 (2.1) | t = 1.64 | 0.24 |
|  | Yes | 6 | 0.5 (1.2) |  |  |
| Burning mosquito repellent | No | 43 | 0.7 (2.0) | perm. test = -7.61 | 0.96 |
|  | Yes | 4 | 2.5 (10.7) |  |  |
| Burning candles | No | 39 | 0.9 (2.0) | t = 0.10 | 0.93 |
|  | Yes | 8 | 0.6 (2.1) |  |  |

IQR, interquartile range. Geo. mean, geometric mean. GSD, geometric standard deviation. (Note: 6 0 ppm observations were recorded to 0.000001 ppm for natural log transformation). Perm. test, test statistic of two-sample permutation test (for cell counts < 5).
