# Supplementary material for: Indoor and outdoor fine particulate matter and carbon monoxide concentrations in homes of infants in Nairobi, Kenya
Source: PLOS Glob Public Health. 2026 Apr 6;6(4):e0006202. doi: 10.1371/journal.pgph.0006202 (PMC13052846; doi:10.1371/journal.pgph.0006202)
Supplement: S7 Table — (DOCX) [file pgph.0006202.s007.docx]

**Indoor and outdoor fine particulate matter and carbon monoxide concentrations in homes of infants in Nairobi, Kenya**

**Supporting information**

**S7 Table. Estimated air changes per hour with the corresponding measured 24 h PM_2.5_ (ug/m^3^) and CO (ppm) concentrations in a subsample of 23 homes.**

| ID | Air changes per hour | Air changes per hour model (R^2^) | PM_2.5_ (ug/m^3^) | CO (ppm) |
| --- | --- | --- | --- | --- |
| 1 | 1.57 | 0.93 | 38.67 | 6.10 |
| 2 | 7.47 | 0.98 | 49.09 | 2.20 |
| 3 | 1.22 | 0.99 | NA | 1.60 |
| 4 | 0.62 | 0.99 | 61.75 | 22.50 |
| 5 | 1.73 | 0.92 | 19.34 | 2.20 |
| 11 | 1.23 | 0.99 | 29.72 | 8.20 |
| 14 | 5.20 | 0.96 | 91.33 | 0.60 |
| 16 | 0.88 | 0.99 | 64.18 | 6.60 |
| 19 | 1.22 | 0.99 | 37.42 | 3.70 |
| 20 | 3.35 | 0.99 | 47.68 | 1.80 |
| 22 | 0.29 | 1.00 | 67.08 | 33.90 |
| 23 | 2.87 | 0.96 | 29.49 | 2.10 |
| 28 | 3.54 | 0.98 | 79.82 | 3.30 |
| 32 | 4.15 | 0.98 | 19.28 | 0.30 |
| 33 | 3.32 | 0.99 | 91.15 | 2.40 |
| 35 | 4.13 | 0.97 | 77.40 | 5.40 |
| 36 | 3.02 | 0.99 | 12.84 | 2.20 |
| 37 | 1.82 | 1.00 | NA | 2.30 |
| 38 | 1.34 | 0.99 | NA | 6.00 |
| 43 | 7.73 | 0.91 | 25.86 | 0.30 |
| 46 | 1.61 | 1.00 | 25.81 | 1.80 |
| 47 | 2.11 | 0.99 | 519.57 | 4.10 |
| 48 | 3.68 | 0.99 | 16.80 | 5.20 |
