# Supplementary material for: Indoor and outdoor fine particulate matter and carbon monoxide concentrations in homes of infants in Nairobi, Kenya
Source: PLOS Glob Public Health. 2026 Apr 6;6(4):e0006202. doi: 10.1371/journal.pgph.0006202 (PMC13052846; doi:10.1371/journal.pgph.0006202)

**Indoor and outdoor fine particulate matter and carbon monoxide concentrations in homes of infants in Nairobi, Kenya**

**Supporting information**

**S3 Fig. Scatter plots and Spearman correlations between estimated air changes per hour and natural log indoor PM_2.5_ (top panel) and 24 h mean CO (bottom panel) concentrations in a subsample of 23 homes** (blue lines and grey bands indicate fitted values and standard error of locally estimated scatterplot smoothing [LOESS] fit).


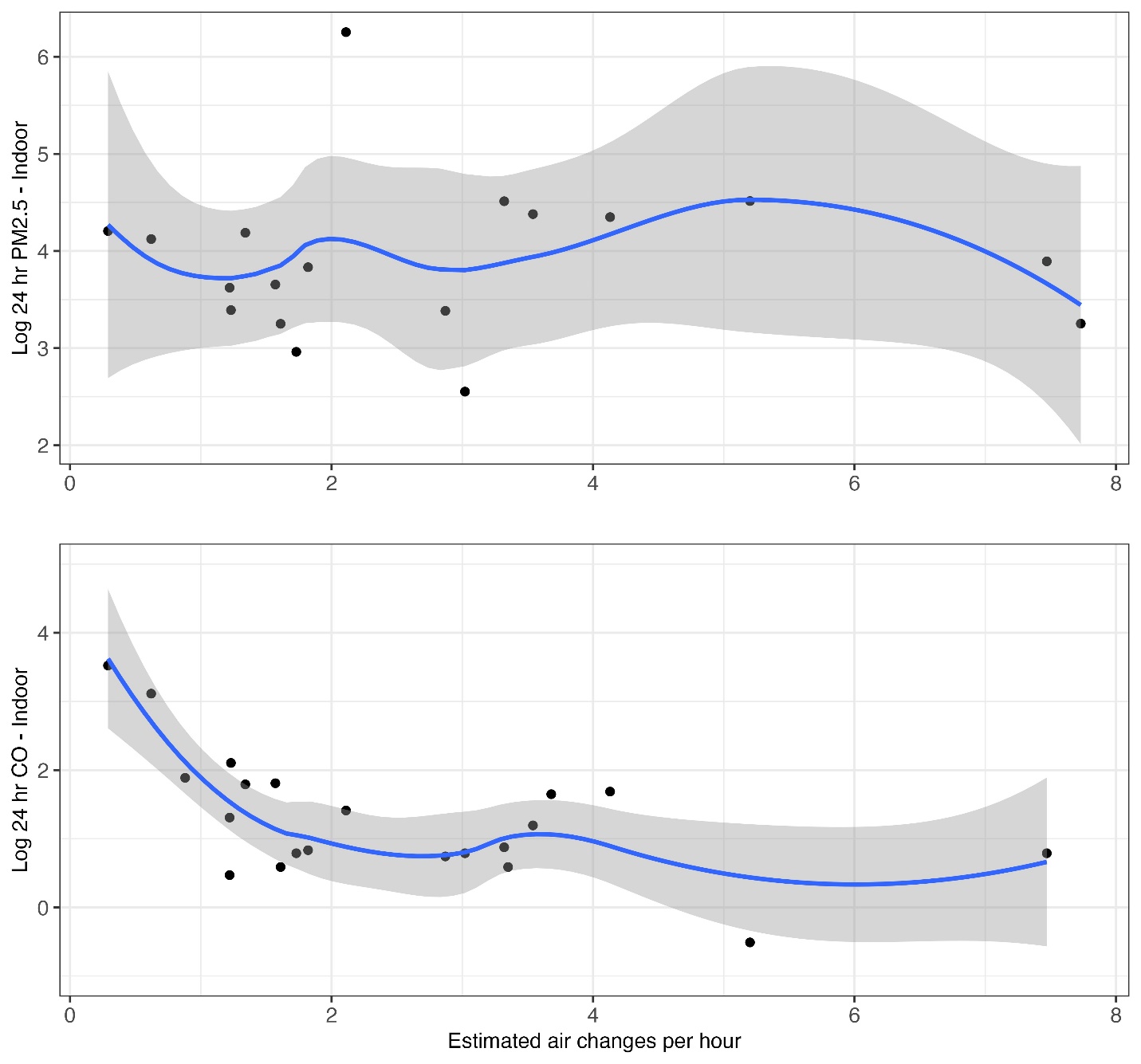

Supplement: S3 Fig — (DOCX) [file pgph.0006202.s010.docx]
