# Supplementary material for: Indoor and outdoor fine particulate matter and carbon monoxide concentrations in homes of infants in Nairobi, Kenya
Source: PLOS Glob Public Health. 2026 Apr 6;6(4):e0006202. doi: 10.1371/journal.pgph.0006202 (PMC13052846; doi:10.1371/journal.pgph.0006202)
Supplement: S1 Checklist — (DOCX) [file pgph.0006202.s011.docx]

**Indoor and outdoor fine particulate matter and carbon monoxide concentrations in homes of infants in Nairobi, Kenya**

**Supporting information**

**S1 Checklist**

**Ethical considerations, permits and authorship**

*This section is applicable to all research types.*

Provide details as to who granted permissions and/or consent for the study to take place in the Methods section of your manuscript. This should include the names of **all** ethics boards, governmental organizations, community leaders or other bodies that provided approval for the study. If individuals provided approval refer to these people by their role or title but do not list their name(s).

Reported on page number: As noted in the Methods section of the manuscript, this study was approved by the Human Subjects Division at the University of Washington, the Kenyatta National Hospital-University of Nairobi Ethics and Research Committee, and Kenya’s National Commission for Science, Technology and Innovation. In addition, permission to recruit participants and conduct study procedures at the Dandora II Health Centre was provided by the Nairobi County Health Services Research Department, the Embakasi North Sub-County, and the Dandora II Health Centre Administration, and following discussion with the Dandora I and II community health workers.

If there were any deviations from the study protocol after approval was obtained please provide details of these changes in the Methods section of your manuscript.

Reported on page number: There were no deviations from the study protocol after approval was obtained.

Did this study involve local collaborators that are residents of the country where the research was conducted or members of the community studied? If you do not have any authors from said communities, please provide an explanation for this below.

Seven of 16 authors are Kenyan and reside in Nairobi, Kenya, where the study is based.

**Human subjects research (e.g. health research, medical research, cross-cultural psychology)**

Did you obtain written informed consent from a representative of the local community or region before the research took place? How did you establish who speaks for the community? Details of written informed consent obtained from study participants should be reported separately in the Methods section of your manuscript.

As described in the Methods section, written informed consent was provided by each study participant. In addition, written permission to recruit participants and conduct the study at the Dandora Health II Centre was granted by the Nairobi County Health Services Research Department, the Embakasi North Sub-County and the Dandora II Health Centre Administration.

How did members of the local community provide input on the aims of the research investigation, its methodology, and its anticipated outcome(s)?

Key stakeholders from research, policy and regulatory networks in both paediatrics and in environmental health participated in a workshop in Nairobi, Kenya in 2019, entitled “Kenya Healthy Home Healthy Brain Project”. Priorities for research and for technology transfer, including a focus on impact of air pollution and other neurotoxicants on neurodevelopment in urban Kenya, and building capacity for blood lead laboratory testing, air pollution sampling and neurodevelopmental assessment were emphasized at the workshop and were incorporated into the study described in this manuscript. Prior to enrolling study participants, the study team met with and received input from staff at the Dandora II Health Centre, including community health workers.

When engaging with the local community, how did you ensure that the informed consent documents and other materials could be understood by local stakeholders?

Informed consent documents and structured interview questions were translated in Kiswahili and submitted to the Kenyatta National Hospital – University of Nairobi Ethics and Research Committee for review and approval. All study staff who interact with participants are fluent in English and Kiswahili and are able to utilize the language of the study participant’s preference.

Will the findings of the research be made available in an understandable format to stakeholders in the community where the study was conducted (e.g. via a presentation, summary report, copies of publications, etc.)? Please provide details of how this will be achieved.

Report back of results and air sampling methods have been shared via presentation at annual dissemination workshops held throughout the study. Workshops have included representatives from the Kenya Ministry of Health regulatory agencies, professional society members, academic institutions and staff working at the facility where the study is based, including community health workers and promoters. The venues for these meetings have included the University of Nairobi and the Dandora II Health Centre, where the study is based.

**Non-human subjects research using specimens/ animals collected as part of the study, or those housed in archival collections. Examples include archaeology, paleontology, botany and zoology.**

Did the permission you obtained from a local authority to perform the study include an agreement on access to outputs and benefit sharing? This may include procedures to enable fair distribution of the benefits and resources arising from the research performed. Please include any details of Prior Informed Consent and Benefit Sharing Agreements obtained. These may be required by field-specific regulations, for example the Convention on Biological Diversity (CBD) and the associated Nagoya Protocol.

N/A

If the material used in your study was imported, please A) provide the year it was imported and B) indicate whether permits were obtained to import/export the materials used, C) provide details of any permits obtained. If this information is not available, please indicate this.

N/A

If you used archival specimens, please state how the material used in your study was acquired by the institute it is held in and provide details of any permits obtained for the original excavations/ sample collection. If this information is not available, please indicate this.

N/A

How was the potential cultural significance of the materials collected in your study to local communities considered in your research design? Were Indigenous peoples and/or local researchers and institutions involved with archaeological excavations / collection of specimens? If so, please provide a description of their involvement.

N/A

If your manuscript includes photographs of human remains please indicate whether authors obtained permission from descendants or affiliated cultural communities to do so.

N/A
