# Supplementary material for: Indoor and outdoor fine particulate matter and carbon monoxide concentrations in homes of infants in Nairobi, Kenya
Source: PLOS Glob Public Health. 2026 Apr 6;6(4):e0006202. doi: 10.1371/journal.pgph.0006202 (PMC13052846; doi:10.1371/journal.pgph.0006202)
Supplement: Attachment — Submitted filename: Kipter et al comments and responses.docx [file pgph.0006202.s014.docx]

**Kipter et al. Revision Comments and Responses**

***Reviewer #1:***

***I have gone through the MS entitled "Indoor and outdoor fine particulate matter and carbon monoxide concentrations in homes of infants in Nairobi, Kenya" thoroughly and found it very interesting. Before publication few changes needed.***

***1. Title of the MS is not representable for the whole study. Please modify as per the main objectives.***

*We appreciate this comment. Per comment #5, we acknowledge that our prior description of the study objectives was overly broad and unclear. We have simplified and clarified the description of the study objectives (see below), and elected not to change the title.*

***2. Add all environmental sample size in methodology section.***

*In methods, line 134, we stated that 48 homes were sampled.*

***3. Also include the how the study came up with the 400 sample size.***

*Thank you for the prompt. We included a citation for the published study protocol, (line 129), which describes the basis of N = 400 in detail. Given that this was based on detecting minimum effect sizes for neurodevelopmental outcomes and given that reviewer 2 suggested omitting reference to neurodevelopmental outcomes, we did not elaborate on the details of our power calculations in the text of the manuscript.*

***4. Conclusion part I feel less informative, please improve.***

Thank you for your comment. We added the percentage of homes exceeding the AQG levels for CO to the Results. In the Discussion, we describe how this compares with other work in SSA, namely Orina et al. 2024, and highlight the need for further studies to examine indoor CO levels in infant and young child homes.

*5.* ***Objectives of the study should be well explain in the MS****.*

We combined and simplified two sentences in the Introduction (last paragraph) to make the main objectives clearer and moved sentences concerning the secondary aims (i.e., using CO decay curves and calculating I/O ratios) to Methods.

***Reviewer #2:***

***Thank you for giving me the opportunity to review this paper. I will give you some opinions and suggestions and hope the manuscript will be improved.***

***[Abstract]***
***1. Although the abstract mentions an assessment of air pollution and neurodevelopmental outcomes in children, the results and conclusions do not address neurodevelopmental outcomes. Since the manuscript does not cover this topic in detail, I recommend removing references to neurodevelopmental outcomes to avoid confusion.***

Thank you for your comment. We removed reference to the longitudinal study design in the Abstract and also removed details about the cohort and study visits from the Methods section.

***2. The authors evaluated exceedances of indoor and outdoor PM2.5 and CO concentrations based on WHO air quality guideline (AQG) levels.***
***However, it is important to note that WHO AQGs primarily focus on outdoor air quality, and more stringent standards may be needed for indoor environments.***
***The authors should consider discussing this limitation in the Discussion section and/or reference appropriate indoor air quality benchmarks.***

We appreciate this insight and have highlighted the lack of an indoor AQG in the Discussion, line 596.

***[Introduction]***
***3. lines 62-65,***
***This sentence is somewhat unclear. It may be helpful to revise it for clarity and conciseness.***
***Both urban and rural children are likely to be exposed to indoor and ambient air pollution, so the contrast being drawn here seems oversimplified****.* We agree. We removed the comparison of rural and urban areas and simply described the importance of both household and ambient air pollution sources for children in urban areas.

***4.lines 77-79.***
***Please specify whether these PM2.5 concentrations are 24-hour, monthly, or annual averages, as this is critical for interpreting the pollution levels.***

Thank you for pointing this out. We modified the statement to specify that the concentrations are 24 h measurements.

***5. I recommend that the authors include the geometric mean (GeoMean) and geometric standard deviation (GeoSD) in Table 3 to better characterize the distribution of the exposure data, which are likely to be right-skewed.***

We added geometric means and SDs for the PM_2.5_ measurements to Table 3. We were not able to calculate these for the CO measurements because of the many 0 values.

***6. Additionally, regarding the PM2.5 measurement for ID 47 (519.57 µg/m³), do the authors consider this to be a valid measurement? It would be helpful to clarify whether this value was verified or assessed for potential measurement error, as it appears to be an extreme outlier.***

We appreciate the opportunity to clarify this measurement. This was considered a valid measurement after rigorous quality assurance, and quality control steps were completed in accordance with standard EPA guidelines. For context, it may help to note that this participant reported burning mosquito repellent during the 24 h period and thus we believe the value to be plausible.

***7. It would be helpful to include a Spearman correlation matrix of indoor/outdoor PM2.5, CO, temperature, and humidity to better illustrate the relationships between air quality and meteorological variables****.*

Kindly note that scatter plots of 24 h mean temperature (°C) and relative humidity (%) vs. 24 h PM_2.5_ (ug/m^3^) and 24 h mean CO (ppm) are included in the Supporting information. These scatter plots have LOESS fit regression lines. Because no visual associations were apparent in these figures, we did not calculate correlation coefficients for the manuscript. However, we provide them below for your reference:

| **Pollutant** | **RH (%) and Temperature** | **rho** | **P_value** |
| --- | --- | --- | --- |
| IN CO (ppm) | IN RH (%) | 0.29 | 0.04 |
| IN CO (ppm) | IN Temperature | 0.17 | 0.25 |
| IN PM_2.5_ (ug/m^3^) | IN RH (%) | -0.01 | 0.95 |
| IN PM_2.5_ (ug/m^3^) | IN Temperature | 0.12 | 0.45 |
| OT CO (ppm) | OT Temperature | 0.36 | 0.02 |
| OT CO (ppm) | OT RH (%) | -0.16 | 0.36 |
| OT PM_2.5_ (ug/m^3^) | OT Temperature | 0.19 | 0.24 |
| OT PM_2.5_ (ug/m^3^) | OT RH (%) | -0.17 | 0.31 |
| IN, indoor. OT, outdoor. rho, Spearman correlation coefficient | | | |

***8. Given the small sample size, conducting statistical tests may be challenging.***
***I recommend that the authors use non-parametric tests instead of t-tests, as these do not assume normality. Additionally, it would be beneficial to discuss this limitation in the Discussion section to acknowledge its potential impact on the results****.*

Thank you for this suggestion. For comparisons with groups that had fewer than 3 observations, we removed any test of comparison. For comparisons with groups having 3-4 observations, we replaced the t-tests with non-parametric permutation tests.

***9. The authors should provide a more detailed explanation of how the 48 participants were selected from the overall cohort.***

Thank you for your suggestion. In Methods, we described that during recruitment, one or two participants were invited and consented each week to participate in the air sampling visits. In the Discussion, we added sentences to acknowledge that the conspicuous nature of the air monitors may have limited acceptability of air monitoring and that the sample of women consenting to air monitoring may have had limited generalizability to the full cohort. In addition, please see our response to #12 below.

***10. It would be helpful to indicate, in the Methods section, how many participants were excluded from the original cohort of approximately 400, and the reasons for their exclusion.***

*Thank you for your comment. In the Methods, we stated there were no pre-specified exclusion criteria for household air monitoring.*

***This information is important for assessing potential selection bias and the generalizability of the findings.***

We agree. Please see our response to comment #9.

***11. If possible, I suggest adjusting the size and position of the WHO AQG label in Figure 1, as it overlaps with some data points, which may reduce the clarity of the figure.***

Thank you for the prompt. We made this change.

***12. The authors should discuss the representativeness of the study population included in the analysis.***

We added a table to Supporting information: “S1 Table. Comparison of selected demographic and household characteristics at enrollment of air sampling participants versus the full ABC cohort,” and summarize the differences in the “Demographic and Household Characteristics” section of Results. We also added sentences to the Discussion highlighting limited overall generalizability to urban Kenyans, given estimated differences in clean fuel access reported in the Kenyan Demographic and Health Survey, 2022.
***13. In addition, potential biases related to the placement of indoor and outdoor air pollution monitors (for PM2.5 and CO) should be considered****.*

Thank you for this comment. To address this concern, we added a statement to the Discussion acknowledging that area monitoring such as ours do not necessarily capture the full range of participant exposures as personal monitoring would have.

***14. For PM2.5, the lack of continuous measurements limits the ability to capture peak exposure periods.***

We agree. We added a sentence to the Discussion describing this as a limitation.

***15. While CO was measured continuously, a notable limitation is the relatively low proportion of valid measurement time within the 24-hour period.***

Kindly note that there were 47 valid indoor CO samples with sample durations ranging from 24-24.2 h, the target 24 h duration. Of the 41 outdoor samples, there indeed were two shorter duration samples (16.1 h and 16.8 h) but these were still two-thirds of the target duration. We did not consider this to be a major limitation so did not further mention it in the discussion.

***[Conclusion]***
*16. lines 510-511*
***"Four homes had higher indoor CO concentrations than the AQGs."***
***As a concluding statement, this may lack clarity and impact.***
***Providing more context (e.g., total sample size, degree of exceedance, or percentage) would strengthen its significance.***

Thank you for this helpful suggestion. To Results, we added "*Four of 47 (9%) of homes had indoor CO concentrations higher than the AQGs…”* to better contextualize this percentage. In the Discussion, we further highlighted the need for expanded studies of indoor CO in infant homes in this context.
