# Supplementary material for: Indoor and outdoor fine particulate matter and carbon monoxide concentrations in homes of infants in Nairobi, Kenya
Source: PLOS Glob Public Health. 2026 Apr 6;6(4):e0006202. doi: 10.1371/journal.pgph.0006202 (PMC13052846; doi:10.1371/journal.pgph.0006202)
Supplement: Attachment — Submitted filename: Air pollution Nairobi infants_rev2_response.docx [file pgph.0006202.s015.docx]

**Indoor and outdoor fine particulate matter and carbon monoxide concentrations in homes of infants in Nairobi, Kenya**

**Revision 2 – Response to Reviewers**

Thank you for giving us the opportunity to respond to this second round of comments from Reviewer #2, reproduced *verbatim* in **bold** (with our responses beneath):

**Reviewer #2: The authors have addressed the concerns I previously raised, but I believe some additional revisions are necessary.**

**(1) In Table 1-2, some items appear to include overlapping responses, for example, Floor material and Fuels used by the household indoors, and I recommend that the authors add notes or footnotes to clarify these overlaps explicitly.**

Thank you for pointing this out. We added a footnote “Total percentages may exceed 100% as categories were not mutually exclusive” to both tables. Additionally, we changed the description of the indoor cooking fuels in Table 1 as “Indoor combustion/burning (cooking fuels)” and the Table 2 description to “Cooking fuel used during air sampling” so it would be more clear that these are two separate variables.

**(2) Lines 496-497 include the sentence "it is plausible that an indoor ~ outdoor AQG", which is unclear and should be rewritten for clarity.**

We reconsidered this sentence. We decided it is indeed confusing and not necessary, so we deleted it.

**(3) In the Conclusion, while I acknowledge the need for further research, the sentence regarding "Four homes ~ similar contexts" should either be deleted or revised, as it does not effectively highlight the study’s conclusions.**

Thank you for this recommendation—we deleted the sentence.

**(4) When it comes to the global context, generally, indoor and outdoor PM and CO levels are higher outdoors, but in regions such as Nairobi or Sub-Saharan Africa, indoor concentrations remain high, which may be attributed to factors such as fuel use, socioeconomic conditions, and cultural practices. I recommend emphasizing in the discussion the importance of improving indoor air quality in relation to these findings and noting that since most activities are conducted indoors rather than outdoors, the potential for greater health burdens may arise.**

Thank you for the recommendation. In response, we added the following sentence to the Conclusion: “These findings highlight the importance of improving indoor air quality, given that caregivers and infants spend much of their time indoors, which increases their exposures and potential health risks.”

**(5) I suggest that the authors consider additional English editing if possible.**

We did another pass at checking English grammar and made 5-6 minor corrections.
